# Biological Activities of Some Isoquinoline Alkaloids from *Fumaria schleicheri* Soy. Will.

**DOI:** 10.3390/plants11091202

**Published:** 2022-04-29

**Authors:** Ramona Păltinean, Irina Ielciu, Daniela Hanganu, Mihaela Niculae, Emoke Pall, Luc Angenot, Monique Tits, Andrei Mocan, Mihai Babotă, Oleg Frumuzachi, Mircea Tămaş, Gianina Crişan, Michel Frederich

**Affiliations:** 1 Department of Pharmaceutical Botany, “Iuliu Haţieganu” University of Medicine and Pharmacy, 400337 Cluj-Napoca, Romania; rpaltinean@umfcluj.ro (R.P.); mocan.andrei@umfcluj.ro (A.M.); mihai.babota@umfcluj.ro (M.B.); oleg.frumuzachi@gmail.com (O.F.); mtbotanica@yahoo.com (M.T.); gcrisan@umfcluj.ro (G.C.); 2 Department of Pharmacognosy, “Iuliu Haţieganu” University of Medicine and Pharmacy, 400010 Cluj-Napoca, Romania; dhanganu@umfcluj.ro; 3 Department of Clinical Sciences, University of Agricultural Sciences and Veterinary Medicine, 400372 Cluj-Napoca, Romania; mihaela.niculae@usamvcluj.ro (M.N.); emoke.pall@usamvcluj.ro (E.P.); 4 Center of Interdisciplinary Research on Medicines, Laboratory of Pharmacognosy, University of Liège, 4000 Liège, Belgium; l.angenot@uliege.be (L.A.); m.tits@uliege.be (M.T.); m.frederich@uliege.be (M.F.)

**Keywords:** *Fumaria scheleicheri* Soy. Will., isoquinoline alkaloids, HPLC-DAD, in vitro anti-cholinesterase, cytotoxic, antioxidant

## Abstract

*Fumaria schleicheri* Soy. Will. is a species belonging to the Papaveraceae family, being widespread in East-Central and Southern Europe. As with numerous other species of the genus, it is used in traditional medicine for the treatment of hepatobiliary and digestive disorders. The aim of the present study consisted of the evaluation of its alkaloid content and the assessment of its in vitro antioxidant, anti-cholinesterase and cytotoxic potential. Total alkaloid content in the composition of the species was quantified by a spectrophotometrical method and they were individually identified and quantified by HPLC-DAD. The antioxidant capacity was investigated by the DPPH and FRAP methods, while the anti-cholinesterase activity was assessed by an adapted Ellman’s method. The in vitro cytotoxic activity was evaluated on BJ human fibroblasts and DLD-1 human colon adenocarcinoma cell lines. Results showed the presence of bicuculline, protopine, chelidonine, stylopine and sanguinarine, among which bicuculline, protopine, stylopine and sanguinarine were quantified, while the antioxidant and anti-cholinesterase assays showed valuable potentials. No cytotoxic effect was observed on BJ cell lines and selective cytotoxicity was expressed towards tumoral cells. In this context, *F. schleicheri* appears as an important medicinal species with significant potential of substitution with the officinal species.

## 1. Introduction

In Europe, almost 60 taxons of the genus *Fumaria* (Papaveraceae) can be found in the spontaneous flora of different countries [1], especially in the Mediterranean basin and in the Eastern and Western parts of the continent [1,2]. Due to the nomenclatural and taxonomic confusion of the categories at the same level, this genus has been successively introduced in the Papaveraceae family and in the Fumariaceae family, being actually included in the Papaveraceae family, Fumarieae tribe [2,3]. Species of the *Fumaria* genus present similar morphological characters; thus, their identification is difficult and laborious [3,4]. 

The *Fumaria schleicheri* Soy. Will. species is widespread in East-Central and Southern Europe [1] and can be identified by small sepals, deep pink corolla and bracts about as long as the erect fruiting pedicels [4]. It is one of seven species included in the *Fumaria* genus that can be found in the Romanian flora and is widespread in the Western and Central regions of Romania [3,5,6]. 

**Figure 1:**
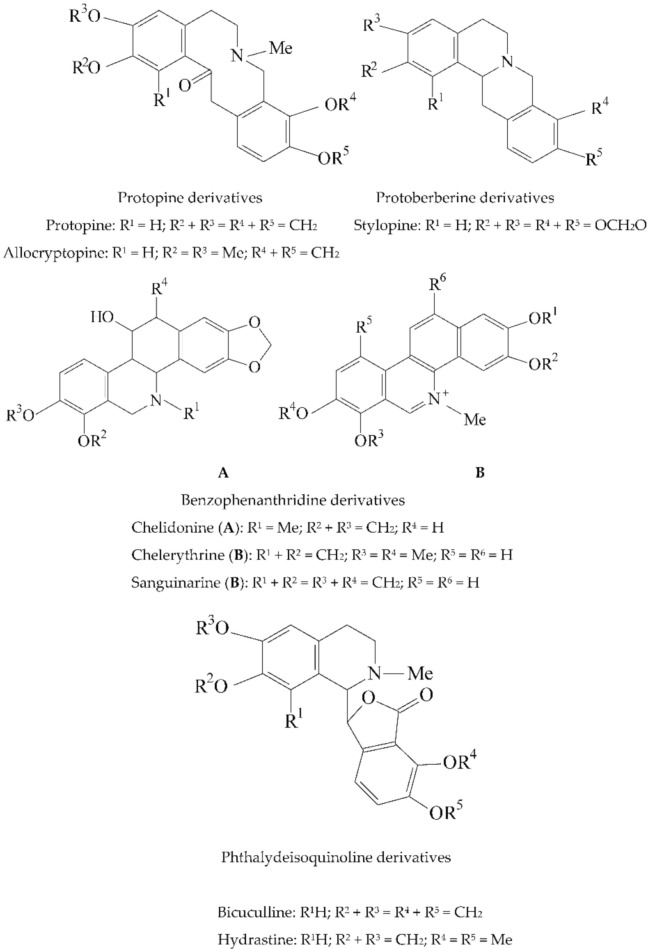
Structures of alkaloids that can be found in the composition of *F. schleicheri* species [11,13,14].

*Fumaria* species have been used since ancient times in folk medicine, especially to treat hepatobiliary and digestive disorders, but also for the treatment of skin and respiratory disorders, rheumatism, hypertension, different types of infections or constipation [7,8,9]. Studies performed on different species of the genus have proven the analgesic, antioxidant, hepatoprotective, antiproliferative, antiplasmodial, antibacterial, antifungal and anti-inflammatory activities of *Fumaria* species [3,8,9,10,11]. These biological activities are attributed to the composition in isoquinoline alkaloids, among which protopine derivatives (e.g., protopine, allocryptopine), protoberberine derivatives (e.g., stylopine), benzophenanthridine derivatives (e.g., sanguinarine, chelidonine, chelerythrine) and phthalyde-isoquinoline derivatives (e.g., bicuculline, hydrastine) (Figure 1) are the predominant classes of compounds [8,9,11,12,13,14]. 

Among the species of the *Fumaria* genus, *F. schleicheri* Soy.-Will. is a species that is lesser studied. The few existing studies cite its anticonvulsant [15,16,17,18] and neuroprotective activities [19], which are assigned also to the composition in alkaloids, such as protopine [17,20], parfumidine, fumaricine, cryptopine, allocryptopine [11,20], stylopine [21] or fumschleicherine [11,20,22]. Other compounds that can be found in the composition of the species in significant amounts are polyphenols, which offer the species antioxidant and diuretic properties [3]. To the best of our knowledge, these studies are the only ones that describe this species from the point of view of its chemical composition and biological activities. 

Cytotoxic activity is reported for species belonging to the *Fumaria* genus, especially for the officinal one [11,23] and it is studied towards various cancerous cell lines (e.g., melanoma, breast cancer, leukemia) [11,23,24,25]. The compounds that are responsible for this activity are the isoquinoline alkaloids [11,14], while the underlying mechanism is related to the antioxidant potential of these compounds [11,23]. 

The acetylcholinesterase inhibitory activity represents the basis of the studies that aim to develop a treatment for Alzheimer’s disease. *Fumaria* species represent one of the numerous genera of plants that have been studied for this biological activity. *F. vaillantii* Loisel [11,26,27], *F. kralikii* Jord [28], *F. indica* Lam [29] and *F. officinalis* L. [14] proved to be the most efficient ones. 

The antioxidant activity is one of the biological activities that is highly studied for the species belonging to the *Fumaria* genus [3,9], being assigned both to their polyphenolic composition [10,30], but also to their alkaloid composition [13,31,32]. It is one of the activities that not only forms the basis of cytotoxic activity [11,23,33], but also the basis of numerous other biological activities, such as hepatoprotective activity [34], antibacterial [13] or antidiabetic one [35]. 

The link between all these concepts represents the purpose of the present study. Therefore, taking all these into consideration, the study of the *F. schleicheri* species and its biological activities appears to be an important subject. In this context, the aim of the present study consisted of the identification and quantification of the isoquinoline alkaloids present in *F. schleicheri* and the evaluation of their biological activities, such as the anti-cholinesterase, antioxidant and cytotoxic ones. In order to evaluate the chemical composition of the species, specimens harvested from different environments were tested. In this way, both the chemical composition of the species and the effect of pedo-climatic conditions on the total alkaloid content could be evaluated. This represents a first argument that sustains the originality of this study, which also consists of the fact that it is one of the few studies existing in scientific literature that aims to bring arguments regarding the cytotoxic, anti-cholinesterase and antioxidant activity of the species and to further confirm these biological activities, which are cited for the alkaloids of the *Fumaria* genus. Moreover, the present study is one of the few studies performed on the species that proves it can be a valuable medicinal product, having important potential for the substitution with the officinal species. 

## 2. Results

Three *F. schleicheri* samples (FS1–FS3) obtained from different Romanian environments were subjected to the phytochemical analysis and antioxidant and acetylcholinesterase inhibitory properties evaluation. The sample with the highest alkaloid content was selected and further investigated for its in vitro cytotoxic potential against both normal and tumoral cells. 

### 2.1. Quantification of the Total Alkaloids Content

The quantification of the total alkaloid content revealed a significant concentration of total alkaloids for all the tested samples (*p* < 0.05) (Table 1). 

It can be clearly observed that the results obtained for the determination of the total alkaloid content highlighted important amounts of alkaloids, with a significantly higher value for the FS3 sample (0.86 ± 0.11) (*p* < 0.05). All samples were collected in the same maturation stages of the species (in May–June, at the complete flowering and fruiting setting stage of the species). It could be observed that the obtained results for all the tested samples depended on the pedo-climatic conditions of the harvested samples, varying accordingly. Results of this quantification of alkaloids represent an element of novelty of the present study, which reports the quantification of this total alkaloid content for the first time. In order to identify the individual alkaloids that are present in the chemical composition of the species, total alkaloids determination was completed by the HPLC-DAD quantification of individual alkaloids from each sample. 

### 2.2. Identification and Quantification of Alkaloids from F. schleicheri Extracts by HPLC-DAD

In the composition of the tested extracts, the presence of alkaloids such as bicuculline, protopine, stylopine, chelidonine and sanguinarine could be observed (Figures S1–S3). Identification of these compounds could be performed by comparison of the obtained results for the tested extract with the ones obtained in the same conditions for commercially available references. 

Identification of individual compounds was achieved by HPLC-DAD and highlighted the presence of bicuculline, protopine, chelidonine, stylopine and sanguinarine. The obtained parameters for each compound (UV spectra) and the retention times (R_t_) were compared to the ones obtained for commercially available references and allowed us to confirm the presence of these compounds in the composition of the tested samples (Table 2). 

Results of the quantification of individual alkaloids showed important amounts especially in the case of protopine, stylopine, bicuculline, chelidonine and sanguinarine (Table 3). In particular, FS1 sample contained the highest amount of stylopine, bicuculline and sanguinarine. Furthermore, for all tested extracts, these amounts were proved to be in direct correlation with the amount of total alkaloids obtained in the spectrophotometrical assays.

### 2.3. Antioxidant Activity of the F. schleicheri Extract

The results obtained using the DPPH and the FRAP assays are presented in Table 4. Important antioxidant properties were observed for all the tested samples, with the most relevant potential exhibited by the FS1 sample for the FRAP (*p* < 0.05) and DPPH (values statistically not significant) assays. 

### 2.4. Anti-Cholinesterase Activity of the F. schleicheri Extract

**Figure 2:**
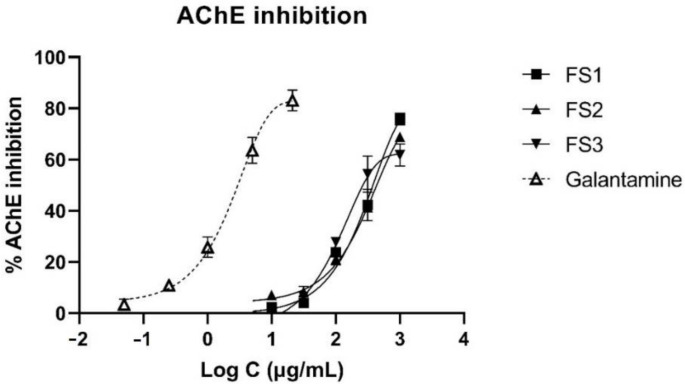
Achetylcholinesterase (AChE) inhibition by *F. schleicheri* extracts and galantamine as reference inhibitor.

Inhibitory potential of alkaloid fractions or individual alkaloids isolated from different *Fumaria* species on acetyl- and butyrylcholinesterase was indicated by several studies [14,26,36]. In this regard, dry extracts of *F. schleicheri* were tested in vitro for their ability to inhibit acetylcholinesterase using Ellman colorimetric assay adapted to a microplate reader method. Inhibition was expressed as IC_50_ (μg/mL) using galantamine as positive control (IC_50_ = 2.00 ± 0.47 μg/mL). All tested samples exerted a medium inhibitory activity, while FS3 proved to be the most active against acetylcholinesterase (*p* < 0.05) (Table 5, Figure 2). 

### 2.5. In Vitro Cytotoxic Activity of the F. schleicheri Extract

**Figure 3:**
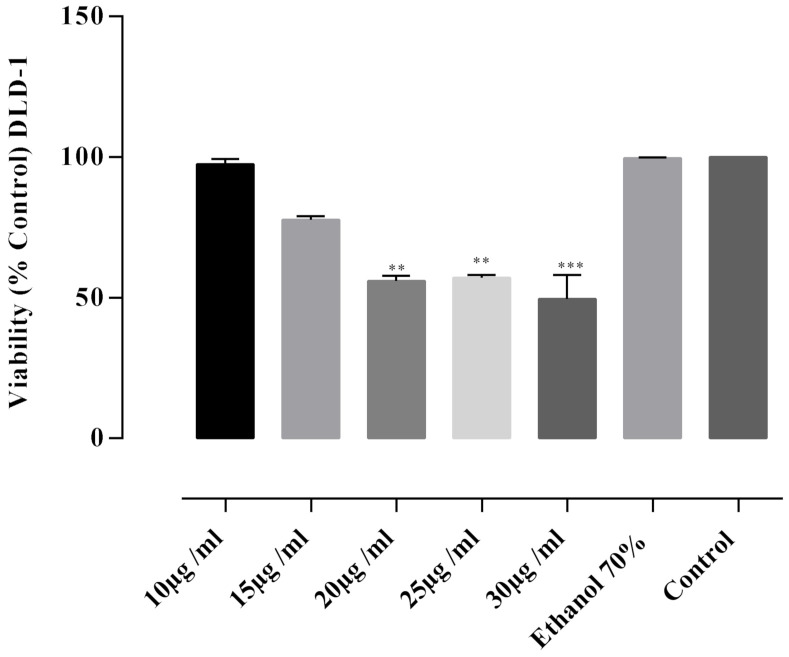
Cytotoxic activity assay results of *F. schleicheri* (FS3) extract on DLD cell line. The extract was tested at five different concentrations 10–30 µg/mL. Negative control—untreated cells. Internal control—cells treated with 70% ethanolic solution. Values represent the mean ± SD of three independent evaluations; ** *p* < 0.001; *** *p* < 0.0001.

**Figure 4:**
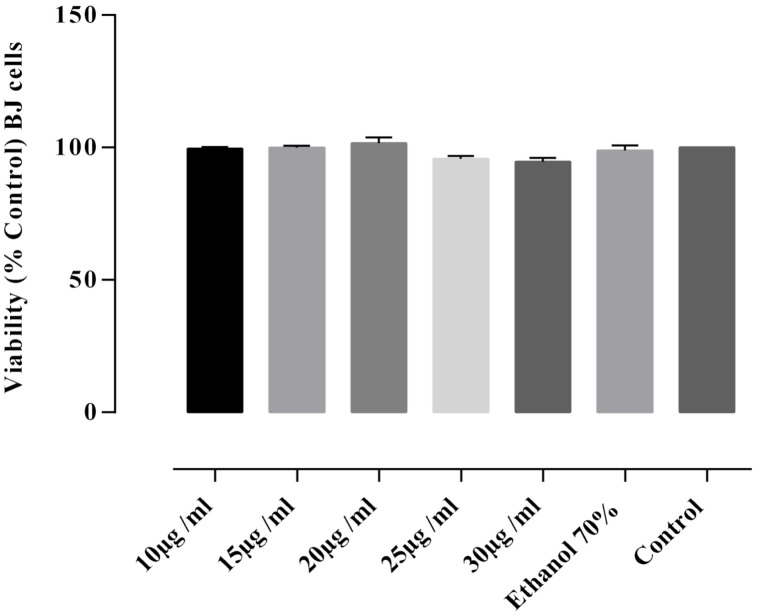
Cytotoxic activity assay results of *F. schleicheri* extract (FS3) on BJ cell line. Negative control—untreated cells. Internal control—cells treated with 70% ethanol solution. Values represent the mean ± SD of three independent evaluations.

For the evaluation of the in vitro cytotoxic activity, the FS3 sample was selected, as it was proved to have the highest alkaloids content. In order to evaluate its inhibitory activity, DLD-1 and BJ cells were incubated with five different concentrations of the extract. In DLD-1 cells the extracts caused inhibitory activity in a dose-dependent manner (Figure 3). A significant increase in the cytotoxic activity *p* < 0.0001) compared to the untreated control was revealed at a concentration of 30 µg/mL extract followed by concentrations of 20 and 25 µg/mL, respectively. The cytotoxic potential was also indicated by calculating the required concentration that inhibited 50% of the DLD-1 cell line (IC_50_ in µg/mL). The concentration of extract required to reduce DLD-1 cell viability by 50% was 21.91 ± 0.38 μg/mL at 24 h. At a concentration of 15 μg/mL, the percentage of cell viability was 77.69 ± 1.26% and 97.53 ± 1.81% at the concentration of 10 μg/mL. At the same concentrations, the average viability of BJ cells was 99.68 ± 0.57% (Figure 4) with no statistically significant (*p* > 0.05) differences between the viability values determined by these concentrations and the negative control. Thus, the tested extract of *F. schleicheri* was considered non-cytotoxic on BJ cells. Selective cytotoxicity was expressed only towards tumoral cells.

## 3. Discussion

*F. schleicheri* is a Papaveraceae species that has received lesser attention in scientific studies along time. However, it is one of the most widespread species in the *Fumaria* genus [3]. In this context, its study appears important, especially as the ethnopharmacological potential of *Fumaria* species is largely known [9]. and the existing studies prove the possibility to use its vegetal medicinal products as substitutes for the officinal species [16,17,18,19]. 

The total alkaloid content was assessed spectrophotometrically hereby for the first time for the *F. schleicheri* species. Other similar studies cite the total quinolizidine contents, which were of 0.43% for *F. capreolata* and 0.52% for *F. bastardii* [37], which proved to be similar with the amount of isoquinoline alkaloids quantified here. Suau et al. assessed the total alkaloids from the aerial parts for *F. sepium* at 0.88% and at 0.83% for *F. agraria* [38]. However, these studies do not use spectrophotometrical methods, which represents therefore an element of novelty of the present study. The only existing study which cites similar results is, to the best of our knowledge, also performed by our team on the officinal species and showed similar amounts of total alkaloids [39]. The sample that showed superior results compared to the for *F. schleicheri* is the FS3 sample, which was also chosen for the cytotoxicity assays. 

The chemical composition of the 70% aqueous ethanolic extract was further investigated using a HPLC-DAD method and compounds were identified by comparison of the obtained parameters with the results obtained for commercially available references that were tested in the same conditions. Among the compounds that were identified, only protopine [11,17,20] and stylopine [21] were previously identified in the composition of the species. For the other compounds, to the best of our knowledge, it is the first scientific evidence of their presence in the composition of the species growing in Romania. The presence of bicuculline, chelidonine and sanguinarine is therefore an element of novelty that this study presents (Table 2). They are valuable compounds, important for the biological activities of this species, that were previously reported in the composition of other *Fumaria* species [7,9,12,39], but not in the composition of *F. schleicheri*. Results obtained in the phytochemical analysis of the *F. schleicheri* extract not only represent elements of originality and are reported for the first time hereby but are also related to the biological activities of the species.

The antioxidant capacity of *F. schleicheri* is reported hereby for the first time, being assigned to its alkaloids content. Other existing studies on the same species assigned this activity to the composition in polyphenolic compounds [3,10]. DPPH and FRAP assays were used to establish the total antioxidant capacity and showed important results for this species. For the officinal species, this activity was assigned to the alkaloid’s composition [13]. The attribution of the antioxidant activity to alkaloids represents therefore another element of novelty of the present study and becomes even more important as this activity seems to represent the basis of numerous other pharmacological activities [13].

The results obtained for the acetylcholinesterase (AchE) inhibitory activity can be explained based on the different distribution of each individual alkaloid in the analyzed samples. In a previous study focused on the evaluation of anti-cholinesterase potential of several *Fumaria* species from Turkey, Sener et al. [36] noticed that this activity varied in a dependent manner by the content in three main alkaloids, the most important being protopine. The most recent studies showed also that the anticholinesterase potential of isoquinolinic alkaloid-rich fractions can be influenced both by the type and amount of each individual compound through a synergistic effect. For example, Tuzimski et al. [40] highlighted a strong in vitro synergistic effect for the pairs protopine-sanguinarine and protopine-chelerythrine which exerted inhibition rates over 97% in comparison with galantamine. Moreover, anti-cholinesterase potential of an alkaloid extract obtained from *F. schleicheri* was evaluated for the first time in the present work, this representing one of the main original elements of the present study. 

A significative negative correlation was noticed between the values obtained for the total alkaloid content and the anti-cholinesterase activity (Pearson correlation coefficient −0.97, *p* < 0.05), indicating that this therapeutic activity depends on the alkaloid concentrations. Given the interpretation of the AchE Inhibition based on the IC_50_ (µg/mL) values, the results suggest a most intense activity with an increasing alkaloid content.

Treatment of BJ cells with 10–30 μg/mL concentration of the extract, did not express a cytotoxic effect (Figure 4). On the other side, the same concentrations of the tested extract, proved selective cytotoxicity towards the human colon adenocarcinoma DLD-1 cell line. This pharmacological activity was previously reported for the officinal species [23,24], being assigned to the isoquinoline alkaloids [14,23]. The in vitro cytotoxic potential of the *F. schleicheri* species adds novelty and originality to the present study, as it is pointed out hereby for the first time, alongside with the other biological activities. 

All these biological activities were previously cited and evaluated for the *F. officinalis* species [9]. Taking into consideration that a significant cytotoxic, anti-cholinesterase and antioxidant properties are revealed in the present study for the *F. schleicheri* species and also the fact that the studied species has an important ethnopharmacological background, it appears that *F. schleicheri* may be taken into consideration for further studies that may highlight the possibility of replacing the vegetal medicinal product with vegetal medicinal products obtained from *F. schleicheri*. 

## 4. Materials and Methods

### 4.1. Chemicals and Reagents

Stylopine and chelidonine were purchased from ChromaDex Inc. (Los Angeles, CA, USA), sanguinarine chloride hydrate and chelidonine from Sigma-Aldrich (Darmstadt, Germany), protopine from Extrasynthese (Genay, France) and bicuculline from Cayman Chemical Company (Ann Arbor, Michigan, USA). All references were of analytical standard. Trifluoroacetic acid for HPLC, HPLC grade acetonitrile and methanol were purchased from Sigma-Aldrich (Darmstadt, Germany). The ultra-pure water used for HPLC analysis was obtained from a Millipore system (Milli-Q RG) (Millipore, France). Acetycholine for the antispastic assays was obtained from Sigma-Aldrich (Darmstadt, Germany). Ferric chloride, 6-hydroxy-2,5,7,8-tetramethylchromane-2-carboxylic acid (Trolox) (97%); diammonium 2,2′-azino-bis(3-ethylbenzothiazoline-6-sulfonate) (ABTS) (>98%), 2,2-diphenyl-1-(2,4,6-trinitrophenyl) hydrazine (DPPH), 2,4,6-tris (2-pyridyl)-striazine (TPTZ) for antioxidant assays, acetylthiocholine iodide (ATCI), acetylcholinesterase from *Electrophorus electricus* (electric eel) (AchE), 5,5′-dithiobis(2-nitrobenzoic acid) (DTNB) and galantamine hydrobromide for the AchE inhibition assay were purchased from Sigma-Aldrich (Darmstadt, Germany).

### 4.2. Plant Material

Aerial parts of *Fumaria schleicheri* Soy. Will. were collected from its natural habitat at the flowering and fruit setting stages. The specimens were obtained from different Romanian environments (Table 6, Figures S4–S6) and identified by the Department of Botany, Faculty of Pharmacy, University of Medicine and Pharmacy “Iuliu Haţieganu” Cluj-Napoca, Romania and deposited at the herbarium of the department (Voucher no. 28.3.3.6-9) [12,39]. 

The fresh aerial parts of plants material were mechanically cleaned and left to dry at 23 °C for 14 days. The dried herbal material was crushed with a Retsch grinder at 350 µm and preserved in the dark at 20 °C [41]. 

### 4.3. Extraction Technique

An amount of 1 g air-dried aerial parts of *Fumaria schleicheri* Soy. Will was mixed with 15 mL of diluted ammonia and extracted with ethyl acetate (3 × 30 mL). The solvent was evaporated to dryness in a rotary evaporator at 40 °C under reduced pressure. The residue was taken up with 100 mL sulfuric acid 0.05 M. After homogenization, the aqueous acid solution was adjusted to pH 9–10 with concentrated ammonia and extracted with ethyl acetate (3 × 30 mL). The extracts were dried over anhydrous sodium sulphate and the solvent was evaporated to dryness in a rotary evaporator at 40 °C under reduced pressure. The residue was dissolved in methanol and transferred into a 10 mL volumetric flask. The solution was diluted to volume with the same solvent and then filtered through 0.45 µm membrane before use. Then, 10 µL of the solution was injected for HPLC-DAD [42,43,44,45,46]. 

### 4.4. Preparation of Standard Solutions and Sample Preparation for HPLC-DAD

Reference standards were diluted in methanol to achieve a concentration of 1 mg/mL. All solutions were stored in a refrigerator at 4 °C prior to analysis. Then, 10 µL of each solution was injected for HPLC-DAD [12,39,42].

### 4.5. Quantification of Total Alkaloid Content

The quantification of total alkaloid content was performed using a method described in the *Chelidonii herba* monograph in the European Pharmacopoeia, 10th edition. The colorimetric quantification assay was carried out using chromotropic acid, which reacts with the formaldehyde released by the methylenedioxy groups of the isoquinoline alkaloids in an acidic medium to form the stable, colored, dibenzoxanthylium cation, which can be revealed by spectrophotometry at 570 nm. The total alkaloids content was calculated using the following formula: A × 2.23/m, considering A the absorbance at 570 nm and m the mass of vegetal powder. Results were expressed as g chelidonine/100 g vegetal product (%). All experiments were performed in triplicate (n = 3) [39,47]. 

### 4.6. HPLC-DAD Conditions

Chromatographic analysis was performed on an Agilent 1100 HPLC system equipped with a binary pump, an autosampler, a column compartment and a UV-VIS diode array detector. Samples were separated on an Inertsil Phenyl RP column (5 µm, 4.6 × 250 mm, Phenomenex, Torrance, CA, USA). The column temperature was set to 25 °C. The mobile phase was composed of a gradient of acetonitrile (A) and 0.1 M triethylamine and 0.01 M sodium hepthanesulphonate, adjusted with H_3_PO_4_ to pH 2.5 (B). The gradient program was set as it follows: 0–1 min 85% B; 1–20 min 70% solution B; 20–30 min 50% solution B, 30–35 min 40% solution B, 35–40 min 85% solution B. The flow rate was kept constantly at 1 mL/min and the injected volume was 10 µL. The UV detection of alkaloids and their references were performed at 210, 240and 290 nm for protopine, stylopine, bicuculline and chelidonine and at 280 and 330 for sanguinarine [12,39]. 

### 4.7. Antioxidant Activity Assays

Preliminary evaluation of in vitro antioxidant potential of the *F. schleicheri* extract was made using two complementary assays (DPPH and FRAP). In the DPPH assay, 30 L of sample was mixed with 270 μL of DPPH solution (0.004% in absolute methanol). The reaction mix was incubated at room temperature in a dark place for 30 min; after incubation, the absorbance of each sample was read at 517 nm. In the FRAP assay, a reaction mixture containing 25 μL of sample and 175 μL of FRAP reagent—acetate buffer (0.3 M, pH 3.6); 2,4,6-tris(2-pyridyl)-s-triazine (TPTZ) (10 mM) in 40 mM HCl; and ferric chloride (20 mM) in a ratio of 10:1:1 (*v*/*v*/*v*)—was incubated for 30 min at room temperature, followed by the reading of the absorbance at 593 nm. For both assays, the results were expressed as mg Trollox equivalents/g dry extract (mg TE/g d.e) [43,45,46].

### 4.8. Anti-Cholinesterase Activity Assays

The extracts were supposed to serial dilutions (10, 31.5, 100, 315, 1000 μg/mL in 50 mM Tris–HCl, pH = 8 containing 5% DMSO) and tested for acetylcholinesterase (AChE) inhibitory activity using an Ellman’s method adapted for 96-wells microplate reader. First, 25 μL of the sample, 50 μL of Tris-HCL buffer (pH = 8, 50 mM), 125 μL of DTNB (0.9 mM in the same buffer) and 25 μL of AchE (0.078 U/mL in same buffer) were mixed in each well and incubated for 15 min at room temperature in a dark place. After incubation, 25 μL of ATCI (4.5 mM in Tris-HCl buffer) were added to each well and incubated again for 10 min. The absorbance of the samples was measured at 405 nm and IC_50_ values (μg/mL) were expressed using galantamine as positive control [46,48].

### 4.9. Cytotoxic Activity Assessment

The cytotoxicity assay of *F. schleicheri* ethanolic extract (FS3) was performed using human fibroblasts BJ (ATCC^®^ CRL-2522™) and human colon adenocarcinoma cell line DLD-1 (ATCC^®^ CCL-221™). The cells were cultured according to standard conditions. The potential cytotoxicity of the tested extract was assessed with (4,5-dimethylthiazol-2-yl)-2,5-diphenyltetrazolium bromide (MTT) assay. To obtain cell suspensions, the cells were treated with 0.25% trypsin-EDTA, and after centrifugation (1500 rpm for 5 min), 1 × 10^4^ cells/well were seeded on 96 wells plates in 200 µL complete culture medium. After 24 h, the *F. schleicheri* extract in five different concentrations (10–30 µg/mL) was added. Negative control samples were represented by untreated cells. The internal control was represented by cells treated with 70% ethanol. Each experimental condition was performed in triplicate. Cell proliferation analysis was performed after 24 h. After 24 h, the medium was removed and 100 µL of 1 mg/mL MTT solution (Sigma-Aldrich, St. Louis, MO, USA) was added. After 3 h of incubation at 37 °C in dark, the MTT solution was removed from each well and 150 µL of DMSO (dimethyl sulfoxide) solution (Fluka, Buchs, Switzerland) was added. Spectrophotometric readings at 450 nm were performed with a BioTek Synergy 2 microplate reader (Winooski, VT, USA). Cytotoxicity was expressed as viability percentage (%) based on the absorbance ratio between cell cultures treated with extract and the negative controls (untreated cells) multiplied by 100. Additionally, the concentration required to inhibit 50% of the cell line (IC_50_ in µg/mL) was calculated from the dose–response curve obtained using non-linear regression [49,50,51]. 

### 4.10. Statistical Analysis

Statistical analysis was performed using ANOVA GraphPad Prism software, version 6.0 (San Diego, CA, USA). The results were presented as mean ± standard deviation (SD). One-way analysis of variance (ANOVA) was conducted, followed by Tukey’s post hoc test, to determine statistical significance. The Pearson correlation analysis was performed to determine the correlation between the total alkaloids content in tested extracts and the anti-cholinesterase activity. A *p* value lower than 0.05 was considered statistically significant.

## 5. Conclusions

Phytochemical analysis of *Fumaria schleicheri* Soy. Will. highlighted the presence of several isoquinolinic alkaloids, namely bicuculline, chelidonine and sanguinarine, which were not previously reported in the composition of the species. Furthermore, the extracts belonging to the aerial parts of this species displayed valuable in vitro antioxidant, anti-cholinesterase and cytotoxic activities. In this way, the species proved to have important potential to be considered in the future as a promising substitute for the officinal species belonging to the *Fumaria* genus. Further studies are necessary in order to establish the mechanisms of action for the biological activities, but the antioxidant activity may represent an important basis for these activities. 

## Figures and Tables

**Table 1:** The total alkaloid content of the tested *F. schleicheri* samples.

Sample	Total Alkaloid Content (g Chelidonine/100 g Vegetal Product %)
FS1	0.52 ± 0.23 ^b^
FS2	0.60 ± 0.03 ^b^
FS3	0.86 ± 0.11 ^a^

Note: Values represent the mean ± standard deviations of three independent measurements. Superscript letters indicate statistically significant differences between the total alkaloid content of the tested samples (*p* < 0.05).

**Table 2:** Alkaloids identified in the tested *F. schleicheri* extracts.

Compound	Retention Time R_t_ (min)	UV λ_max_ (nm)
Protopine	24.79 ± 0.14	210, 290
Bicuculline	22.40 ± 0.04	200, 230
Chelidonine	26.29 ± 0.08	200, 290
Stylopine	27.25 ± 0.10	200, 290
Sanguinarine	29.13 ± 0.03	280, 330

Note: Values represent the mean ± standard deviations of three independent measurements.

**Table 3:** Quantification of alkaloids in the tested *F. schleicheri* samples.

Compound (mg/100 g Dry Weight)	FS1	FS2	FS3
Protopine	191.08 ± 0.56	217.49 ± 0.02	126.45 ± 0.11
Stylopine	24.59 ± 0.20 ^a^	<LoQ	3.55 ± 0.02 ^b^
Bicuculline	67.16 ± 0.55 ^a^	3.77 ± 0.03 ^c^	26.84 ± 0.20 ^b^
Sanguinarine	5.61 ± 0.04 ^a^	2.11 ± 0.03 ^b^	1.67 ± 0.016 ^c^
Chelidonine	<LoQ	<LoQ	<LoQ

Note: Values represent the mean ± standard deviations of three independent measurements. <LoQ—identified, but not quantified (below quantification limit). The superscript letters indicate statistically significant differences between the quantified alkaloids values (*p* < 0.05).

**Table 4:** Results for the antioxidant activity assays for the tested *F. schleicheri* samples.

Sample	DPPH (mg Trollox Equivalents/g Dry Extract)	FRAP (mg Trollox Equivalents/g Dry Extract)
FS1	38.93 ± 0.59	68.03 ± 0.46 ^a^
FS2	32.50 ± 1.35	39.30 ± 0.13 ^c^
FS3	29.07 ± 1.36	50.65 ± 0.65 ^b^

Note: Values represent the mean ± standard deviations of three independent measurements. The superscript letters indicate statistically significant differences between antioxidant activity of the tested samples (*p* < 0.05).

**Table 5:** Results of the anti-cholinesterase activity assays for the tested *F. schleicheri* samples.

Sample	AchE Inhibition IC_50_ (µg/mL)	Galantamine IC_50_ (µg/mL)
**FS1**	456.72 ± 50.63 ^b^	2.00 ± 0.47
**FS2**	381.83 ± 102.90	
**FS3**	349.24 ± 51.42 ^a^	

Note: Values represent the mean ± standard deviations of three independent measurements. The superscript letters indicate statistically significant differences between the anti-cholinesterase activity of the tested samples (*p* < 0.05).

**Table 6:** The collected *F. schleicheri* samples and their harvesting place.

Sample	Harvesting Place
FS1	Deva. Hunedoara County
FS2	Târgu Mureş, Mureş county
FS3	Nădăşel, Cluj county

## Data Availability

Data sharing not applicable.

## Supplementary Materials

The following supporting information can be downloaded at: https://www.mdpi.com/article/10.3390/plants11091202/s1, Figure S1. HPLC-DAD chromatogram of the FS1 sample, Figure S2. HPLC-DAD chromatogram of the FS2 sample, Figure S3. HPLC-DAD chromatogram of the FS3 sample, Figure S4. FS1 sample during the harvesting stage, Figure S5. FS2 sample during the conditioning phase, Figure S6. FS3 sample during the drying phase.
