# Supplementary material for: Biological Activities of Some Isoquinoline Alkaloids from Fumaria schleicheri Soy. Will
Source: Plants (Basel). 2022 Apr 29;11(9):1202. doi: 10.3390/plants11091202 (PMC9105361; doi:10.3390/plants11091202)
Supplement: Supplementary file 1 [file plants-11-01202-s001.zip › plants-1700752-supplementary.pdf]

plants

MDPI

Supplementary Materials

Biological activities of some isoquinoline alkaloids from *Fumaria schleicheri* Soy. Will.

Ramona Păltinean [1,#], Irina Ielciu [1,*], Daniela Hanganu [2], Mihaela Niculae [3], Emoke Pall [3], Luc Angenot [4], Monique Tits [4], Andrei Mocan [1], Mihai Babotă [1,#], Oleg Frumuzachi [1], Mircea Tămaş [1], Gianina Crişan [1] and Michel Frederich [4]

[1] Department of Pharmaceutical Botany, "Iuliu Haţieganu" University of Medicine and Pharmacy Cluj-Napoca, 400010, Romania; rpaltinean@umfcluj.ro (R.P.); mocan.andrei@umfcluj.ro (A.M.); mihai.babota@umfcluj.ro (M.B.); oleg.frumuzachi@gmail.com (O.F.); mtbotanica@yahoo.com (M.T.); gcrisan@umfcluj.ro (G.C.)
[2] Department of Pharmacognosy, "Iuliu Haţieganu" University of Medicine and Pharmacy, Cluj-Napoca 400010, Romania; dhanganu@umfcluj.ro (D.H.)
[3] Department of Clinical Sciences, University of Agricultural Sciences and Veterinary Medicine Cluj-Napoca, 400374 Romania; mihaela.niculae@usamvcluj.ro (M.N.); emoke.pall@usamvcluj.ro (E.P.)
[4] Center of Interdisciplinary Research on Medicines, Laboratory of Pharmacognosy, University of Liège, 4000, Liège, Belgium; l.angenot@uliege.be (L.A.); m.tits@uliege.be (M.T.); M.Frederich@uliege.be (M.F.)

* Correspondence: irina.ielciu@umfcluj.ro (I.I.)
\# These authors contributed equally to this work

S. Supplementary materials – Figures S1-S3

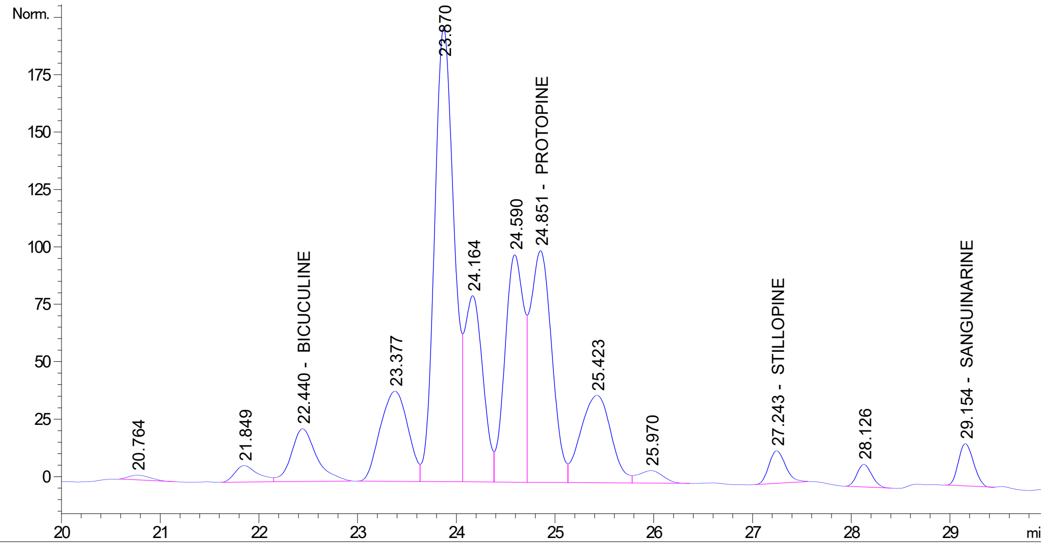

Figure S1. HPLC-DAD chromatogram of the FS1 sample

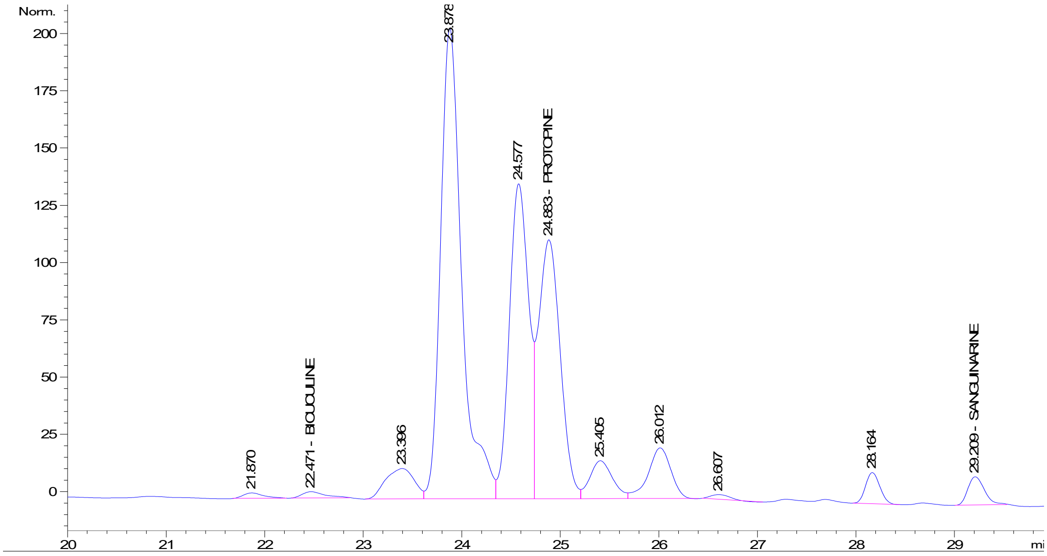

Figure S2. HPLC-DAD chromatogram of the FS2 sample

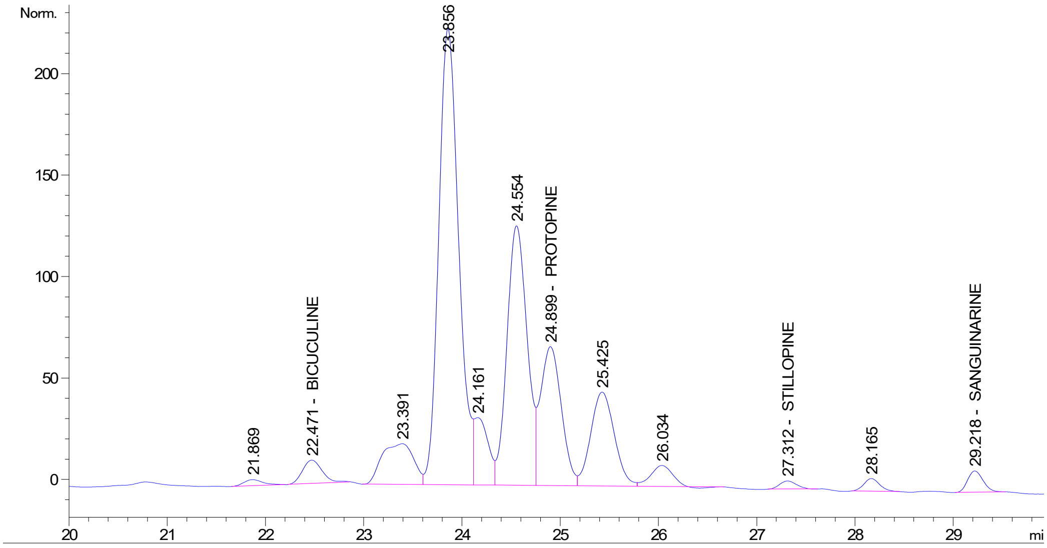

Figure S3. HPLC-DAD chromatogram of the FS3 sample

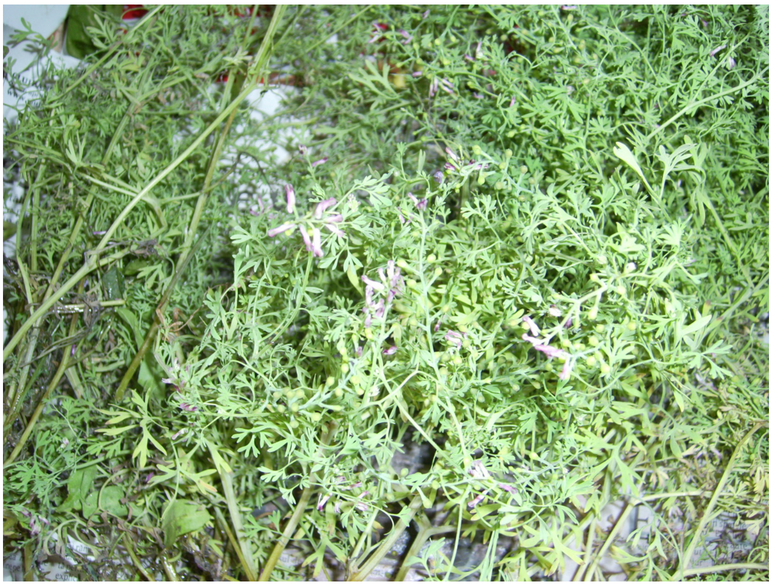

Figure S4. FS1 sample during the harvesting stage

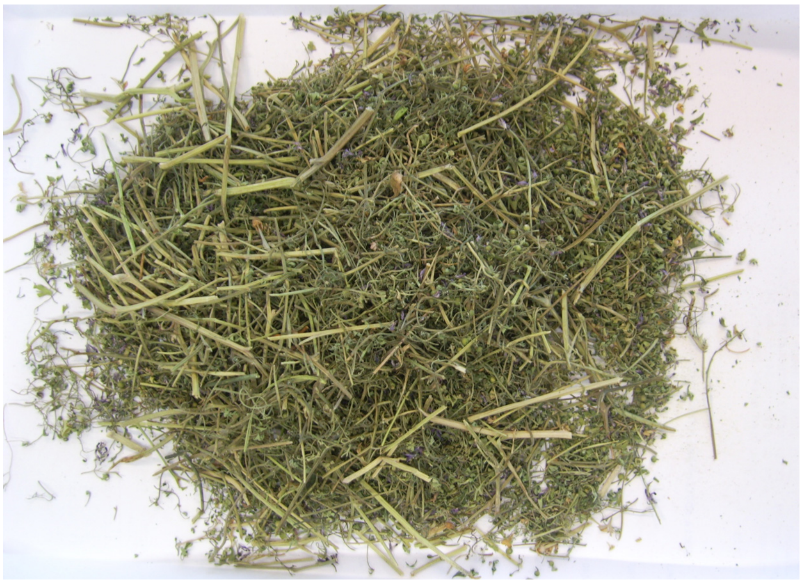

Figure S5. FS2 sample during the conditioning phase

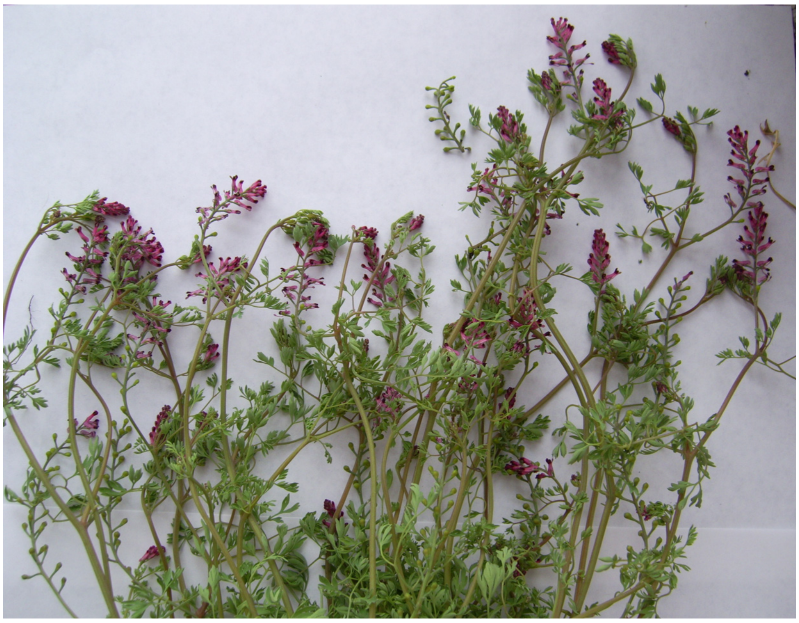

Figure S6. FS3 sample during the drying phase
